# Adolescent religious attendance and spirituality—Are they associated with leisure-time choices?

**DOI:** 10.1371/journal.pone.0198314

**Published:** 2018-06-18

**Authors:** Klara Malinakova, Andrea Madarasova Geckova, Jitse P. van Dijk, Michal Kalman, Peter Tavel, Sijmen A. Reijneveld

**Affiliations:** 1 Olomouc University Social Health Institute, Palacky University Olomouc, Olomouc, Czech Republic; 2 Department of Community and Occupational Medicine, University Medical Center Groningen, University of Groningen, Groningen, The Netherlands; 3 Graduate School Kosice Institute for Society and Health, Pavol Jozef Safarik University in Kosice, Kosice, Slovak Republic; 4 Department of Health Psychology, Faculty of Medicine, Pavol Jozef Safarik University in Kosice, Kosice, Slovak Republic; 5 Institute of Active Living, Faculty of Physical Culture, Palacky University Olomouc, Olomouc, Czech Republic; 6 Department of Social Medicine and Public Health, Faculty of Medicine and Dentistry, Palacky University Olomouc, Olomouc, Czech Republic; Harvard Medical School, UNITED STATES

## Abstract

**Background:**

Spirituality and religious attendance (RA) have been associated with personal attitudes and values, and this may affect lifestyle. The aim of this study was to explore their association with adolescent leisure-time choices in a highly secular environment.

**Methods:**

A nationally representative sample of adolescents (*n =* 4,182, 14.4±1.1 years, 48.6% boys) participated in the 2014 Health Behaviour in School-aged Children cross-sectional study. We measured RA, spirituality (adjusted shortened version of the Spiritual Well-Being Scale), excessive television, computer games, and internet use, as well as participation in organized leisure-time activities.

**Results:**

Compared to non-attending and non-spiritual respondents, respectively, both attending respondents and spiritual respondents were less likely to watch television and play computer games excessively, with odds ratios (ORs) ranging from 0.6 (95% confidence interval 0.5–0.8) to 0.92 (0.9–0.99). Only attending and only spiritual respondents were more likely to use the internet excessively, but this was not the case for those that were both attending and spiritual. Moreover, religious and spiritual respondents were more likely to be involved in at least one organised activity. ORs were 2.9 (1.9–4.3) for RA and 1.3 (1.2–1.4) for spirituality compared to their counterparts. The same pattern was observed for sporting and non-sporting activities combined (ORs 4.6 (3.0–7.1) and 1.5 (1.4–1.7), respectively) and regularly reading books or playing a musical instrument.

**Conclusions:**

Adolescent RA and spirituality are associated with a more active way of spending leisure-time. Further research should focus on understanding potential mechanisms that underlie these associations.

## Introduction

Recently, the amount of time spent on screen-based activities (SBA) has emerged as an important and independent risk factor for the physical and mental health of children and adolescents [1]. Excessive amounts of SBA have been shown to be associated with overweight [2], unfavourable levels of several cardiovascular risk factors [3] as well as a higher occurrence of headache and irritability and reports of feeling low and nervous [4]. Some of the content of SBA seems to add to these risks. For example, playing violent computer games or watching violent television programs were linked to aggressive thoughts, hostility and less pro-social behaviour [5, 6]. Higher levels of screen-based sedentary behaviours have also been linked to other health damaging behaviours, such as substance use [7].

A current criterion for excessive adolescent screen-based activity is spending more than two hours a day on recreational screen time [8], but most adolescents exceed this limit. Moreover, screen time is growing in North America and Europe [9], including the Czech Republic [10]. The fact that sedentary behaviour tracks from childhood into adulthood [11] highlights the need to address this issue in adolescence and to support healthier alternatives for adolescent leisure-time choices.

Organised leisure-time activities (OLTA) are sometimes mentioned as a healthy alternative for SBA. They have also been associated with other positive outcomes, such as lower substance use [12], better school performance and attachment to school [13] and better physical and mental health [14]. Several factors are known to be associated with adolescent participation in OLTA, such as parental support of the activity, friends, self-efficacy, academic achievement, psychopathological problems and environmental factors [15–18]. However, it seems that adolescents themselves associate their involvement in structured leisure activities especially with their intrinsic motivation [17].

Religiosity and spirituality could be of special interest in leisure choices, because they are connected with many dimensions of human life and personal values [19, 20] and also comprise both an organization of norms and behavioural expectations that can lead to a preference for certain activities above others [21]. Thus far, this potentially important group of determinants has not often been studied, and if it has, it has been mostly done in the United States, in which a significant segment of the population identifies with a religious institution. In contrast, the Czech Republic is the country with the highest percentage (76.4%) of people that do not have a religious affliation in the world [22], meaning religion is not a major determinant of main stream youth culture. This makes it a unique population for research in this field, enabling the specific effect of religion to be established apart from only that of main stream youth culture.

Therefore, the aim of this study is to assess the relationship between religious attendance and spirituality (both separately and jointly) and leisure-time choices, specifically SBA and OLTA, among adolescents in a highly secular environment. For the purpose of this article, *spirituality* is understood as internal individual contentedness, one’s perceived closeness to God and one’s sense of meaning of life and of spiritual well-being [23].

## Methods

### Participants and procedure

We obtained data on a nationally representative sample of Czech boys and girls from the 2014 Health Behaviour in School-aged Children (HBSC) study. This cross-sectional WHO collaborative study focused on health and health-related behaviour and their socioeconomic determinants in 11-, 13-, and 15-year-old children. The HBSC study has been conducted at 4-year intervals since 1983/84 and now includes 44 countries across Europe and North America [24]. According to the HBSC study protocol, schools were selected randomly after stratification by region, school size and type of school (primary schools vs. secondary schools). Out of 243 contacted schools 242 schools agreed to participate (response rate 99.6%). Then, classes from the 5^th^, 7^th^ and 9^th^ grades, in general corresponding to age the categories of 11-, 13- and 15-year-olds, were selected at random, one from each grade per school. Data from 14,539 pupils were obtained (response rate 89.2%). The majority of non-response was due to illness or other reasons, e.g. sports or academic competitions (10.6%), and 30 children refused to participate in the survey (0.2%).

Data were collected between April and June 2014. Questionnaires were distributed by trained administrators with no teachers present in the classroom in order to reduce response bias. Respondents had one school lesson (45 minutes) dedicated to completing the questionnaire. The spirituality questionnaire was offered to only half of the adolescents from the 7^th^ and 9^th^ grades, so for the purpose of this paper the dataset included 4,889 adolescents who filled out this section. Of these, 707 were excluded because of incomplete information on age, gender, spirituality or religious attendance, or because of an age outside of the intended age-bracket, i.e. 12.5 to 16.4 years. This led to a final sample of 4,182 respondents (mean age = 14.4, SD = 1.1, 48.6% boys).

Participation in the survey was anonymous and voluntary. The Czech HBSC study was conducted under the auspices of the Ministry of Education, Youth and Sports of the Czech Republic and the World Health Organization Country Office in the Czech Republic. The study design was approved by the Ethics Committee of the Faculty of Physical Culture, Palacky University in Olomouc (No. 17/2013), and conducted in accordance with the ethical requirements formulated by the Convention on Human Rights and Biomedicine (40/2000 Coll.). Other information regarding the ethical issues connected with this study can be found in the study of Badura, Sigmund [13], which dealt with the same primary data.

### Measures

*Religious attendance* was measured as the frequency of attending church or religious sessions using the question: “How often do you go to church or to religious sessions?” Possible answers were: several times a week; approximately once a week; approximately once a month; a few times a year; or never. Those who reported attending religious sessions at least once a week were considered *attending*.

*Spirituality* was measured using the adjusted shortened version of the Spiritual Well-Being Scale (SWBS) [25] measuring overall spiritual well-being. Response possibilities for all seven items regarded a 6-point scale that ranged from ‘strongly agree’ (1) to ‘strongly disagree’ (6), leading to scores from 7 to 42. A higher score represented greater spiritual well-being. In the analyses, spirituality was used as a continuous variable, but for the purpose of dichotomisation for sensitivity analysis, participants with a score of 34 or higher (the upper quartile of the scores) were considered as spiritual, and the rest as non-spiritual. Cronbach’s alpha was 0.81 in our sample.

SBA was assessed using three variables: excessive use of television, the internet, and computer games. *Excessive television use* was assessed by the question: “About how many hours a day do you usually watch television (including YouTube and similar pages), a DVD or similar programs on a screen in your free time?” with nine response categories ranging from ‘I do not watch at all’ to ‘About seven or more hours a day’. Following the HBSC dichotomisation [26], watching television for two or more hours per day on weekdays was classified as excessive.

*Excessive playing of computer games* was measured with the question: “About how many hours a day do you usually play games on a computer, games console, tablet (e.g. iPad), smartphone or other electronic device (do not count physical fitness games) in your free time?” with nine response categories ranging from ‘not at all’ to ‘about seven or more hours a day’. Following the HBSC dichotomisation [26], playing computer games two or more hours on weekdays was classified as excessive.

*Excessive internet use* was measured with the Excessive Internet Use scale [27], which assesses the frequency of five behaviour symptoms of excessive internet use (“I felt uncomfortable when I could not be on the internet.”; “I found myself surfing the internet, even though I did not enjoy it.”; “I neglected my family, friends, school work or hobbies because of the time spent on the internet.”; “I tried to reduce the time spent on the internet, but without success.”), with responses being: Very often / Often / Sometimes / Almost never. ‘Often’ and ‘Very often’ in any of the items were classified as using the internet excessively.

*Participation in organized leisure-time activities (OLTA)* was assessed by the question: “In your free time, do you do any of these organized activities?” with the explanation: “We mean activities you do in sports or other clubs or organizations” followed by six items dealing with different types of leisure-time activities (team sports, individual sports, art school, youth organizations, activities in leisure-time centres and church meetings or singing), including country-specific examples. The possible answers were ‘Yes’ or ‘No’. For the purpose of a more detailed analysis, the respondents clustered as follows: 1) Not active (not involved in a sporting or a non-sporting activity); 2) Active only in sports; 3) Active only in non-sporting activity; 4) Active in both sporting and non-sporting activity.

*Moderate-to-vigorous physical activity (MVPA)* was measured with the question: “Over the past 7 days, on how many days were you physically active for total of at least 60 minutes per day?”with the introductory instruction: “Physical activity is any activity that increases your heart rate and makes you get out of breath some of the time“, which was followed by a few examples of possible kinds of physical activity. According to the WHO recommendation [28], the participants who reported being physically active 7 days in a week were considered as having a sufficient MVPA while the remaining participants as not having a sufficient MVPA.

*Additional leisure time activities* were assessed by the question: “In your free time, how often do you devote yourself to the following activities?”followed by the concrete specifications of the activities (reading books, playing a musical instrument, creative activities) with five response categories ranging from ‘Daily’ to ‘Never’. ‘Daily’ and ‘A few times a week’ were classified as practicing the activity regularly, with the rest classified as non-regular.

*The socioeconomic status* of the respondents' families was used as a covariate and was assessed by The Family Affluence Scale (FAS) [29]. The scale examines the number of cars owned by the family, having one's own bedroom, number of computers in the household, number of family holidays outside of the country, number of bathrooms, and dishwasher ownership. The summary score ranges from 10 to 13 and following HBSC recommendations it was converted into a fractional rank (ridit) score, leading to transformation of ordinal data to an interval scale with a normalised range (from 0 to 1, with higher score indicating higher socioeconomic position) and distribution.

*Perceived family support* was used as a covariate and was measured using the Multidimensional Scale of Perceived Social Support (MSPSS) family subscale [30], which is assessed with four items. Response options ranged from 1 (very strongly disagree) to 7 (very strongly agree). For the purpose of the analysis, a mean MSPSS score was used.

### Statistical analyses

First, we described the background characteristics of the sample. We then assessed the associations of religious attendance (Model 1) and spirituality standardized to z-scores (Model 2) separately, their combination (Model 3) and their interaction (Model 4) with three types of screen-based activities using a binary logistic regression model adjusted for gender, age, socioeconomic status and perceived family support. Each of the independent variables was assessed in a separate model. In the same way we assessed the associations of religious attendance and religiosity with the OLTA; first the associations with the binary overall OLTA variable (at least one activity vs. inactive) using a binary logistic model were assessed, and next the associations with the various OLTA clusters were examined using a multinomial logistic regression model. In the last step we used a binary logistic model to assess the associations of religious attendance and spirituality with the selected additional leisure time activities.

We repeated the analyses with spirituality as dichotomised instead of as a continuous variable, leading to similar results. Therefore, we used the dichotomised variable for the graphical representation of the associations with screen-based activities and OLTA. In the tables, however, we present only the results of analyses with the continuous variable. All analyses were performed using the statistical software package IBM SPSS version 21.

## Results

### Description of the population

The background characteristics of the sample are presented in Table 1. Of the respondents, as measured here, 7.1% were religiously attending, and 9.1% were spiritual, i.e. scored in the highest quartile of the spirituality scale. Religious attendance and spirituality were moderately correlated (r = 0.4).

**Table 1:** Description of the characteristics of the study population.

	Number	%
Gender		
Boys	2,034	48.6
Girls	2,148	51.4
Age		
13 years old (7^th^ grade)	2,091	50.0
15 years old (9^th^ grade)	2,091	50.0
Religious attendance		
Attending (≥ 1/week)	296	7.1
Non-attending (< 1/week)	3,886	92.9
Spirituality		
Spiritual (score 34–42)	399	9.5
Non-spiritual (score 7–33)	3,783	90.5
Screen-based activities^a^		
Excessive television use	2,519	60.5
Excessive computer games playing	1,746	42.1
Excessive internet use	1,297	31.7
Participation in each OLTA separately^b^		
Team sports	1,880	45.0
Individual sports	1,131	27.0
Elementary art school	1,218	29.1
Children and youth organisations	452	10.8
Activities in leisure-time centres	654	15.6
Church activities	271	6.5
OLTA clusters		
Not active	930	22.2
Active: only sports	1,403	33.5
Active: only other activity	772	18.5
Active: sport + other activity	1,077	25.8
Additional leisure time activities^c^		
Sufficient physical activity	786	18.9
Regular reading of books	1,335	32.1
Regular playing musical instrument	827	20.0
Regular creative activities	737	17.8
Total	4,182	100

Notes: Number of missing cases per variable: Religious attendance—0; spirituality—0; Excessive television use—16; Excessive computer games playing- 35; Excessive internet use—86; Organised activities– 65; Sufficient physical activity—27; Regular reading of books—18; Regular playing of a musical instrument -38; Regular creative activities– 65.

^a^Only the respondents with the occurrence of the excessive behaviour are presented.

^b^Only the active respondents are presented

^c^Only the respondents with the occurrence of the activity are presented.

### Screen-based activities

**Fig 1:**
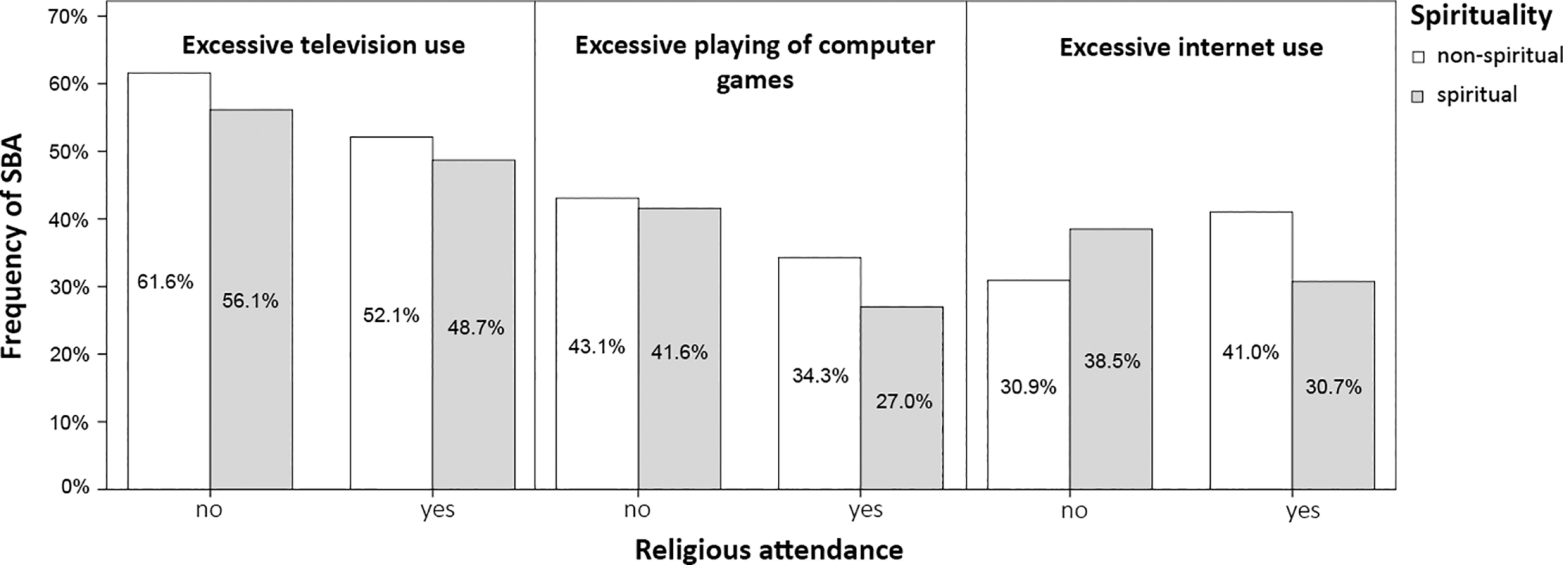
**Frequency of adolescent excessive television use, excessive computer games playing and excessive internet use with dichotomised spirituality and religious attendance**.

Table 2 shows the associations of screen-based activities with religious attendance and spirituality. Both attending (Model 1) participants and spiritual (Model 2) participants were less likely to report excessive use of television and computer games. Moreover, in the case of excessive playing of computer games, a significant interaction showed that religious attendance reinforced the association of spirituality with this behaviour. We found no significant associations of excessive internet use with religious attendance or spirituality separately, or in their combination (Model 3). However, their interaction (Model 4) was associated with a significantly lower likelihood of excessive internet use among participants who were both attending and spiritual (or non-attending/non-spiritual) compared with those who either only attended or were more spiritual. For a graphical representation of sensitivity analysis of the interaction using the dichotomised spirituality variable, see Fig 1.

**Table 2:** Associations of adolescent excessive television use, excessive computer games playing and excessive internet use with religious attendance and spirituality^a^.

	Excessive television use		Excessive computer games playing		Excessive internet use	
	n (%)	OR (95% CI)	n (%)	OR (95% CI)	n (%)	OR (95% CI)
**Model 1: Religious attendance only**						
Non-attending	2,370 (61.2)	**1**	1,657 (43.0)	**1**	1,193 (31.4)	1
Attending	149 (50.3)	0.6 (0.5–0.8)***	89 (30.5)	0.6 (0.4–0.7)***	104 (35.6)	1.2 (0.9–1.5)
**Model 2: Spirituality only (per SD)**		0.92 (0.9–0.99)*		0.9 (0.8–0.96)**		1.04 (0.97–1.1)
**Model 3: Religious atendance and spirituality combined**						
Attending vs. Non-attending		0.7 (0.5–0.9)**		0.6 (0.5–0.8)**		1.1 (0.8–1.5)
Spirituality (per SD)^b^		0.96 (0.9–1.03)		0.94 (0.9–1.01)		1.03 (0.95–1.1)
**Model 4: Interaction**						
Attending vs. Non-attending		0.7 (0.5–1.1)		0.8 (0.6–1.3)		1.5 (0.997–2.2)
Spirituality (per SD)		0.97 (0.9–1.05)		0.96 (0.9–1.03)		1.1 (0.97–1.1)
Religious attendance x Spirituality (per SD)		0.9 (0.7–1.1)		0.8 (0.6–0.999)*		0.8 (0.6–0.998)*

Notes

*p<0.05

**p<0.01

***p<0.001

^a^All the associations were adjusted for age, gender, socioeconomic status (FAS) and family support (mean MSPSS).

^b^Spirituality (per SD) = spirituality score standardized to z-scores

### Organised leisure-time activities (OLTA)

Most adolescents were involved in at least one of the six types of organized activities, the average number of activities being 1.3 (SD = 1.1) in the total sample. Attending respondents participated on average in 2.3 (SD = 1.3) different activities, while non-attending in 1.3 (SD = 1.0) (*p*<0.001). The rate of participation of attending respondents was higher in all observed non-sporting activities (*p*<0.001). Regarding participation in sporting activities, no significant differences were observed among the groups.

**Fig 2:**
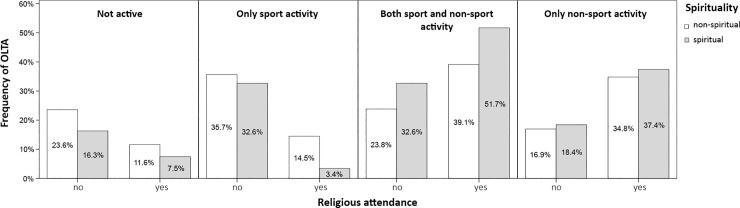
Associations of adolescent OLTA clusters with dichotomised spirituality and religious attendance.

The results of binary logistic regression using the dichotomised overall OLTA variable showed that both attending respondents and spiritual respondents were more likely to be involved in at least one OLTA, with OR = 2.9 (1.9–4.3) for religious attendance and OR = 1.3 (1.2–1.4) for spirituality (*p*<0.001). There were no statistically significant interactions of religious attendance and spirituality. See Fig 2 for a graphical representation.

We next performed multinomial logistic regression analyses with the inactive cluster being the reference category (Table 3), which showed that both attending and being spiritual tended to have more non-sporting activities or a mixture of both sporting and other activities (Fig 2). The attending respondents were approximately two-times less likely to be involved exclusively in sporting activities, but they did not differ significantly regarding general participation in such activities (not shown).

**Table 3:** Association of participation in various types of organised leisure time activities (OLTA) with religious attendance and spirituality^a^.

	Only sports activity		Only non-sports activity		Both sports and non-sports activities	
	n (%)	OR (95% CI)	n (%)	OR (95% CI)	n (%)	OR (95% CI)
**Model 1: Religious attendance**						
Non-attending	1,376 (35.4)	1	666 (17.1)	**1**	944 (24.3)	**1**
Attending	27 (9.1)	0.6 (0.4–1.05)	106 (35.8)	5.3 (3.5–8.2)***	133 (44.9)	4.6 (3.0–7.1)***
**Model 2: Spirituality only (per SD)**		1.1 (1.04–1.3)**		1.4 (1.3–1.6)***		1.5 (1.4–1.7)***
**Model 3: Religious atendance and spirituality combined**						
Attending vs. Non-attending		0.5 (0.3–0.9)*		4.0 (2.5–6.3)***		3.1 (2.0–4.8)***
Spirituality (per SD)^b^		1.2 (1.1–1.3)**		1.2 (1.1–1.4)***		1.4 (1.2–1.5)***
**Model 4: Interaction**						
Non-attending (vs. Attending)		0.8 (0.4–1.5)		2.9 (1.6–5.3)**		3.0 (1.7–5.2)**
Spiritual (vs. Non-spiritual)		1.2 (1.1–1.3)***		1.2 (1.1–1.3)**		1.4 (1.2–1.5)***
Attending x Spiritual		0.6 (0.4–1.002)		1.4 (0.9–2.00)		1.1 (0.7–1.5)

Notes:

*p<0.05

**p<0.01

***p<0.001

^a^All the associations were adjusted for age, gender, socioeconomic status (FAS) and family support (mean MSPSS).

^b^Spirituality (per SD) = spirituality score standardized to z-scores

### Additional leisure-time activities

Table 4 shows the associations of selected adolescent leisure-time activities with religious attendance and spirituality. Both attending (Model 1) participants and spiritual (Model 2) participants were more likely to read books and to play a musical instrument; those with a high level of spirituality were more likely to have sufficient physical activity. Regression model was not significant in the case of regular art activities. Similarly, the interaction effect was not significant for any of these variables.

**Table 4:** Associations of selected adolescent leisure time activities with religious attendance and spirituality^a^.

	Sufficient physical activity		Reading books		Playing musical instrument		Regular creative activities	
	n (%)	OR (95% CI)	n (%)	OR (95% CI)	n (%)	OR (95% CI)	n (%)	OR (95% CI)
**Model 1: Religious attendance only**								
Non-attending	733 (19.0)	1	1,194 (30.8)	**1**	702 (18.2)	**1**	674 (17.5)	1
Attending	53 (18.2)	0.99 (0.7–1.3)	141 (48.1)	2.1 (1.6–2.7)***	125 (43.1)	3.4 (2.6–4.4)***	63 (21.6)	1.3 (0.9–1.7)
**Model 2: Spirituality only (per SD)**^**b**^		1.1 (1.02–1.2)*		1.1 (1.01–1.2)*		1.4 (1.3–1.5)***		1.1 (0.98–1.2)
**Model 3: Religious attendance and spirituality combined**								
Attending vs. Non-attending		0.8 (0.6–1.2)		2.1 (1.6–2.7)***		2.6 (1.9–3.4)***		1.2 (0.9–1.7)
Spirituality (per SD)^b^		1.1 (1.04–1.2)**		0.999 (0.9–1.1)		1.2 (1.1–1.3)***		1.05 (0.95–1.1)
**Model 4: Interaction of Religious attendance and spirituality**								
Attending vs. Non-attending		1.2 (0.7–1.8)		2.1 (1.4–3.1)***		2.2 (1.4–3.3)***		1.5 (0.97–2.3)
Spirituality (per SD)		1.2 (1.1–1.3)*		0.999 (0.9–1.1)		1.2 (1.1–1.3)***		1.1 (0.97–1.2)
Religious attendance x Spirituality (per SD)		0.8 (0.6–1.02)		0.99 (1.001–1.3)		1.1 (0.9–1.5)		0.8 (0.6–1.1)

Notes:

*p<0.05

**p<0.01

***p<0.001

^a^All the associations were adjusted for age, gender, socioeconomic status (FAS) and family support (mean MSPSS).

^b^Spirituality (per SD) = spirituality score standardized to z-scores

## Discussion

We found that religious attendance and spirituality separately were associated with a lower prevalence of excessive television use. The same held for excessive playing of computer games, where in addition, religious attendance reinforced the protective effect of spirituality. Regarding excessive internet use, respondents who were either only attending or only spiritual were more likely to use the internet excessively. However, the combination of attending religious activities and being spiritual was protective with respect to excessive internet use.

We further found that attending respondents, as well as spiritual respondents, were more likely to be involved in at least one activity and tended to have a greater variety of OLTA (a combination of sporting and non-sporting activities). They were also more likely to regularly read books and to play a musical instrument. Spirituality was also associated with higher chances of having sufficient physical activity.

We found that both attending respondents and spiritual respondents were less likely to watch television or play computer games excessively, while religiosity and spirituality did not show any significant association with excessive internet use unless they were in interaction. The limited evidence on religiosity and television viewing has yielded contrasting findings [31, 32], and this also holds for excessive internet use [33, 34]. However, recent students among adolescents observed that religious and spiritual youths watched less television and played fewer video games [35–37], which corresponds with our findings. One of the possible explanations regarding our results could be that in families with high religiosity/spirituality parents tend to keep more oversight of adolescent behaviour [38–40]. This may promote internalisation of adult behavioural norms [38]. Thus the parents’ attitudes and behaviour can be a model that shapes adolescent leisure choices. Some parents put a higher emphasis on the positive developmental outcomes of leisure activities [41]. Unstructured activities such as television viewing and playing computer games may be seen as less desirable within families that regularly attend religious activities if the content does not reflect the same or similar value systems.

In our study we observed that attending as well as spiritual respondents were more likely to participate in at least one OLTA, and they tended to participate in a greater variety of activities. In addition, when considering sporting versus non-sporting activities, they were less likely to be involved solely in sports. Moreover, they were more likely to regularly read books and to play a musical instrument. Spirituality was also associated with higher chances of having sufficient physical activity. There are several possible explanations for these results. First, approximately half of the religious and one-third of the spiritual respondents reported being engaged in some kind of church activity, which itself elevated the number of attended activities. Second, given that care for children and their development is seen as a relatively important value in religious families [42], attending various activities as well as reading or playing a musical instrument may be supported by parents who see these activities as promoting child development. Third, within the local religious community, different activities are often offered, including sports [43]. Attending adolescents might be therefore more likely to get a multiple offer of activities of various kinds, which could also explain their lower exclusive involvement in sports. Fourth, religious programs can serve as a natural platform for the development of relationships [44], and peers who are already involved in some activity may represent another motivation for participation in OLTA [45]. Moreover, religious congregations also represent places where adolescents can make significant encouraging contact with other adults [35], which may attract them to some activities. It is therefore possible that religious attendance and sprituality may promote involvement in organised activities via several routes of community belonging.

We further found that respondents who were both attending and spiritual were less likely to use the internet excessively. Moreover, a sensitive analysis with dichotomised spirituality revealed that in contrast respondents who were either only attending or only spiritual more likely to use the internet excessively. This suggests that in our population the respondents who did not have problem with internet overuse were either both attending and spiritual or they were neither of these. An association with an escape motive is commonly mentioned in the case of excessive internet use [46]. Therefore, it is possible that a combination of religious attendance and spirituality could serve as a coping resource which, together with higher social support, could lower the need for escape into a virtual world [37]. At the same time, some research shows that the inconsistency in religiosity and spirituality levels is associated with a higher vulnerability to mental disorders or problematic behaviour [47, 48], which is in line with our results. Moreover, non-attending spiritual participants could be less likely to benefit from social support connected with an organised religion. Therefore, further analyses of separate as well as combined effects of religious attendance and spirituality and different aspects of human behaviour could help us to understand better the underlying mechanisms.

### Strengths and limitations of this study

This study has several important strengths, the most important being the large and representative sample size of adolescents, the high response rate and the use of the well-established HBSC methodology. A limitation is the relatively small number of attending respondents, which may have affected our power to detect differences despite our large sample. However, this sub-sample still included 296 respondents. Another limitation might be our use of adolescent self-report, which can be inaccurate or influenced by social desirability. Given the prevailing secular attitude within the country, this may have led to some underreporting of RA and spirituality, and thus some underestimating of the associations. Regarding SBA, validation studies [47] did not show the tendency to overestimate or underestimate daily amounts. A last limitation is the cross-sectional design of the study, which does not allow us to make conclusions on causality.

### Implications

Our findings reveal that adolescent religious attendance and spirituality are associated with their leisure-time choices. This suggests that future studies should focus on understanding the direction and potential pathways for these relationships. Consequently, it could assess whether educational programs for adolescents aimed at fostering spiritual values could help lower the occurrence of undesirable behaviours.

### Conclusion

To the best of our knowledge, this is the first study examining the associations of SBA, OLTA and adolescent religious attendance and spirituality. We found that both attending respondents and spiritual respondents were less likely to watch television or play computer games excessively. Respondents who were either only attending or only spiritual were more likely to use the internet excessively. However, the combination of attending religious activities and being spiritual was protective with respect to excessive internet use. These respondents were further more likely to be involved in organized activities, tended to participate in a greater variety of them and were more likely to regularly read books and to play a musical instrument. Spirituality was also associated with higher chances of having sufficient physical activity. This suggests that increasing secularisation might lead to further unfavourable changes in adolescent SBA and OLTA.

## Supporting information

S1 Database Adolescent religious attendance and spirituality—Are they associated with leisure-time choices. (SAV)
